# Fat-Free Mass and Skeletal Muscle Mass Gain Are Associated with Diabetes Remission after Laparoscopic Sleeve Gastrectomy in Males but Not in Females

**DOI:** 10.3390/ijerph19020978

**Published:** 2022-01-16

**Authors:** Ngan Thi Kim Nguyen, Nguyen-Phong Vo, Shih-Yi Huang, Weu Wang

**Affiliations:** 1 International Master Program in Medicine, College of Medicine, Taipei Medical University, Taipei 11301, Taiwan; kimngan1702@gmail.com; 2 School of Nutrition and Health Sciences, Taipei Medical University, Taipei 11301, Taiwan; 3 International Ph.D. Program in Medicine, College of Medicine, Taipei Medical University, Taipei 11301, Taiwan; nguyenphongmd@gmail.com; 4 Graduate Institute of Metabolism and Obesity Sciences, Taipei Medical University, Taipei 11301, Taiwan; 5 Nutrition Research Center, Taipei Medical University Hospital, Taipei 110301, Taiwan; 6 Division of Digestive Surgery, Department of Surgery, Taipei Medical University Hospital, Taipei 11301, Taiwan; 7 Department of Surgery, College of Medicine, Taipei Medical University, Taipei 11301, Taiwan

**Keywords:** laparoscopic sleeve gastrectomy, obesity, diabetes remission, fat mass loss, muscle mass

## Abstract

Besides massive body weight loss, laparoscopic sleeve gastrectomy (LSG) causes massive lean mass, including fat-free mass (FFM) and skeletal muscle mass (SM) that present higher metabolic rates in males. This study examines sex differences in FFM and SM changes of type 2 diabetes (T2D) remission at 12 months post-LSG. This cohort study recruited 119 patients (53.7% females) with T2D and obesity (body mass index 42.2 ± 7.0 kg/m^2^) who underwent LSG. Fat-mass (FM) loss was higher in males than in females (−12.8 ± 6.2% vs. −9.9 ± 5.0%, *p* = 0.02) after one-year post-operation. Regardless of the weight-loss difference, males had higher FFM and SM gain than did females (12.8 ± 8.0 vs. 9.9 ± 5.0% *p* = 0.02 and 6.5 ± 4.3% vs. 4.9 ± 6.2%, *p* = 0.03, respectively). Positive correlations of triglyceride reduction with FM loss (r = 0.47, *p* = 0.01) and SM gain (r = 0.44, *p* = 0.02) over 12 months post-operation were observed in males who achieved T2D remission. The T2D remission rate significantly increased 16% and 26% for each additional percentage of FFM and SM gain one year after LSG, which only happened in males. Increased FFM and SM were remarkably associated with T2D remission in males, but evidence lacks for females.

## 1. Introduction

Obesity with type 2 diabetes (T2D) has become a pandemic disease worldwide [1]; by 2035, diabetes is expected to affect 591.9 million people in the world and more than 60% of the Asian population [2]. Noticeably, Asian exhibits severe T2D and obesity-related diseases such as microvascular and macrovascular complications [3] with a relatively low body mass index (BMI) that is prone to public health alterations [4].

Laparoscopic sleeve gastrectomy (LSG) is a popular bariatric surgery due to its efficacy, safety, and low chance of malnutrition. Importantly, LSG results in long-lasting T2D remission in T2D patients with obesity [5,6]. In LSG, most of the gastric fundus along greater curvature is removed, leading to restricted calorie intake. Hence, patients attempt massive weight loss (WL) and dramatic fat mass (FM) loss to manage obesity, reduce hyperlipidemia, improve insulin resistance, and eventually reverse T2D [3,7]. The role of WL on fatty acid metabolism and lipid accumulation within the muscle in the pathogenesis of obesity and T2D was demonstrated, but the precise mechanism is still a great deal of debate [8].

Despite excess WL after bariatric surgery, LSG results in greater lean body mass loss when compared with gastric bypass [9]. A recent longitudinal cohort study determined that fat-free mass (FFM) and skeletal muscle mass (SM) are expected to be decreased with a significant reduction in WL in the first-year post-operation and remained stable after this time point [10]. Though the increased SM prevented T2D development via hyperphagia reduction and glucose homeostasis [11], the association between SM changes and T2D remission after LSG is scarce. In the clinic, a protein-enriched diet is recommended after LSG to minimize the FFM and SM loss that plays a central role in the whole-body protein metabolism, slows the rate of WL, and reduces weight gain [12]. The recent finding showed that SM protected metabolically healthy boys with obesity from cardiometabolic risks, but not girls [13]. Hence, the association of FFM and SM that account for metabolic regulation [14] with T2D remission post-LSG requires an understanding of sex-manner. Therefore, this study investigated the effect of FFM and SM on diabetes remission stratified by sex differences after 12 months of LSG. Our findings may heighten awareness of sex-manner changes in body composition, focusing on FFM and SM concerning diabetes remission after LSG to reduce T2D-related microvascular and macrovascular complications.

## 2. Materials and Methods

### 2.1. Study Design

We conducted a cohort study among Taiwanese patients with T2D and obesity who underwent LSG between 2014 and 2016 at Taipei Medical University Hospital, Taiwan. Obesity was defined as BMI > 30 kg/m^2^ [15], and T2D was referenced to the American Diabetes Association classification [16].

Inclusion criteria for this study were as follows: (1) T2D diagnosis with poor glycemic control under medication; (2) no history of bariatric surgery; and (3) age over 18 years old. Exclusion criteria were (1) pregnancy, (2) acquired end-organ damage, and (3) C-peptide level < 0.2 ng/mL.

The clinical practice guideline for bariatric surgery was applied to support the perioperative nutrition and medications of these patients [17]. In more detail, it consisted of a three-month low-energy formula diet and a followed 2–8-week structured foods, which was suggested to patients. Nutritional counseling was given to patients during hospitalization and at follow-up visits. Patients were universally advised to sustain a hypocaloric, protein-rich diet with 22–25 kcal/ideal body weight as suggested by ASMBS guidelines [17]. Additionally, patients were recommended to have milk protein supplements (93% milk protein, Sentosa Milk Protein-S, Sentosa, Taipei, Taiwan) according to the nutritional counseling provided by dietitians.

### 2.2. Definition of T2D Remission

Complete T2D remission was defined as HbA1c < 5.7% or FBG < 100 mg/dL and no regular T2D medication [18,19]. Only patients who achieved complete T2D remission were considered to have T2D remission. 

### 2.3. Covariates

Anthropometrics and body composition were measured before surgery and at each postoperative follow-up visit. Patients undergoing the analysis were subjected to standardized conditions, i.e., 2 h fasting and no physical exercise 12 h prior to the measurement. Participants were guided to remove all worn jewelry and metal objects and were barefoot and, in their underwear, to estimate body composition using multiple-frequency bioelectrical impedance, the InBody 230 (Biospace, Seoul, Korea) to obtain the body weight, FM, and SM. Height was measured with a portable stadiometer with a precision of 0.1 cm and a range of 1.00–1.99 m (CROWN HGM-300, Taiwan). We calculated weight-related data as follows: BMI = (Bodyweight)/(height^2^) in kg/m^2^; percent WL = (baseline body weight − 12-month body weight)/(baseline body weight); percent FM loss = 12-month FM in percent − baseline FM in percent; percent SM gain = 12-month SM in percent − baseline SM in percent; percent FFM = (1 − FM in percent); and percent FFM gain = 12-month FFM in percent − baseline FFM in percent. 

Participants were asked for 8–10 h fasting and not to engage in prolonged exercise 24 h prior to the blood test. Blood samples were obtained from capillary sampling between 6:00 a.m. and 9:00 a.m. Eight-hour fasting blood samples were obtained to analyze fasting blood glucose (FBG), and HbA1c (Roche Cobas e602). Total cholesterol, triglyceride (TG), and low-density lipoprotein cholesterol (LDL-C) levels were also determined.

### 2.4. Statistical Analysis

Data are presented as the mean ± standard deviation (SD). A paired Student’s *t*-test was used to observe mean differences (MD) of covariates at baseline and 12 months post-LSG. An independent Student’s *t*-test was used to obtain MD of covariates between females and males. Analysis of covariance (ANCOVA), adjusted for age (years), was used to compare the percent of WL, FM loss, FFM, and SM gain at 12 months after LSG. We used multivariate logistic regression to investigate associations between body composition changes and T2D remission 12 months after LSG, after adjustment for sex, age, and baseline FBG. The receiver operating characteristic (ROC) curve and area under the ROC curve (AUC) were used to determine the discriminatory power of FFM and SM gain. The AUC of 0.501–0.699, 0.700–0.799, and 0.800–0.899 were considered poor, moderate, and excellent discrimination, respectively [20]. Spearman correlation was used to assess the association between the changes in body composition and lipid profile changes over 12 months. Results are presented as odds ratio (OR) with 95% confidence interval (CI). Statistical significance was defined as a two-sided *p*-value < 0.05, performed by IBM SPSS Statistics for Windows, version 25 (IBM Corp., Armonk, NY, USA).

## 3. Results

### 3.1. Clinical Characteristics of the Patients before the Surgery

**Figure 1:**
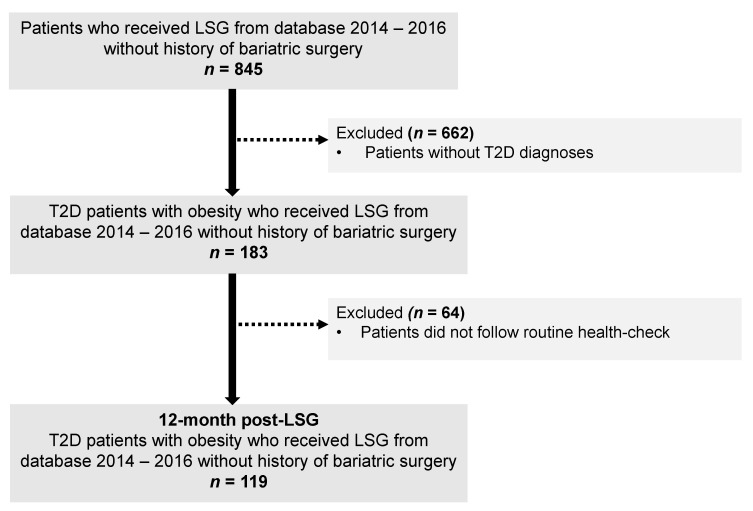
The flowchart of the study. Abbreviation: LSG, Laparoscopic sleeve gastrectomy; T2D, type 2 diabetes.

The flowchart of the study selection process is illustrated in Figure 1. In total, 119 patients were included. These patients had a mean age of 41.9 (9.5) years, mean BMI 42.2 (7.0) kg/m^2^, and over haft of them were female (53.7%). At the initial visit, no significant differences in ages, BMI, HbA1c, and lipid profile between females and males were observed. Compared to females, male patients had a higher percentage of body weight, FFM, SM, and a lower percentage of FM (Table 1). At 12 months after LSG, 40/64 females (62.5%) and 31/55 males (56.4%) patients achieved diabetes remission, and no statistically significant differences between sex and T2D remission rates were observed *(p* = 0.57).

### 3.2. Body Composition Changes at 12 Months after LSG according to Sex Difference

A significant decrease was observed in body weight, FM, FFM, and SM 12 months after LSG. Males experienced a significantly higher reduction in body weight than females (Table 2). The proportion of FFM and SM were also higher in males than in females after 1-year of LSG [FFM: 70.7 (8.4) vs. 61.9 (6.2) %; SM: 39.4 (4.6) vs. 33.7 (3.5)].

### 3.3. Association between Gaining of FFM and SM and Diabetes Remission for 12 Months after LSG in Male Patients

**Figure 2:**
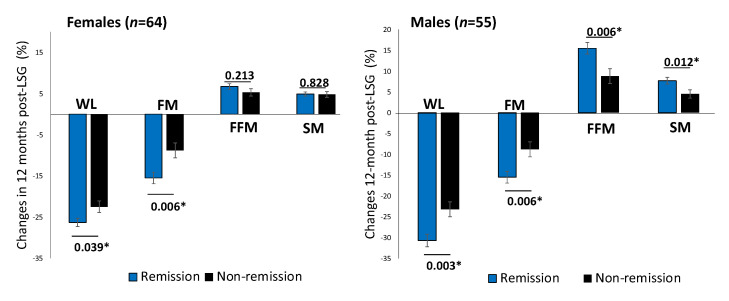
Changes in body composition between T2D remission and non-remission at 12 months post-LSG according to sex difference. Comparison of percent changes of WL, FM loss, FFM, and SM between T2D remission (black bars) and non-remission (grey bars) groups one year after LSG in females (*n* = 64) and males (*n* = 55). The analysis of covariance test was adjusted for age. * *p* < 0.05. Abbreviation: WL: weight loss; FM: fat mass; FFM: fat-free mass; SM: skeletal muscle mass.

One year after surgery, patients achieving diabetes remission showed a significant decrease in BMI (MD, −11.2 (4.4) kg/m^2^, *p* < 0.001). Males with T2D remission showed a significantly higher proportion of FFM and SM increment than those without remission after adjusting for ages baseline FBG (Figure 2).

**Figure 3:**
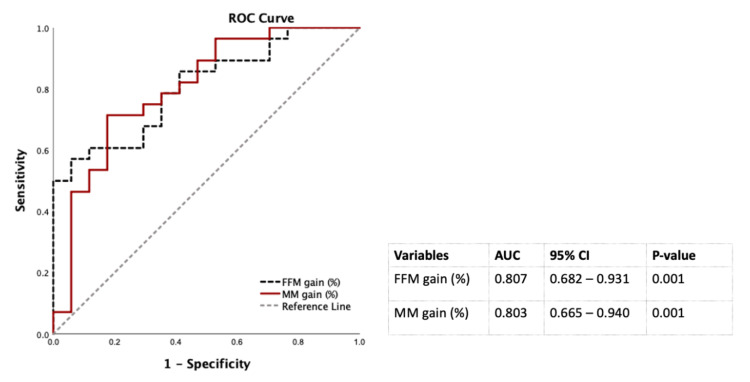
Discrimination power of percentages of FM loss and SM gain 12 months post-LSG using the ROC curves for males. Abbreviation: AUC: area under the ROC curve; CI: confidence interval; FFM: fat-free mass; LSG: laparoscopic sleeve gastrectomy; ROC: receiver operating characteristic; SM: skeletal muscle mass.

Table 3 showed that the WL and FM loss were positively associated with diabetes remission at 12 months after LSG in the multivariate logistic regression analysis controlled by sex, ages, baseline FBG. Of note, each additional percentage of FFM and SM gain, the chance of diabetic remission significantly increased to 16% and 26%, respectively in males, but not in females (Table 3). Therefore, FFM and SM gain to predict diabetes remission at 12 months post-LSG in males were conducted using the ROC curves. The AUCs of FFM and SM gain were 0.807 and 0.803 that were classified as excellent factors to discriminate diabetes remission from non-diabetes remission at 12 months after LSG in males (20) (Figure 3).

**Figure 4:**
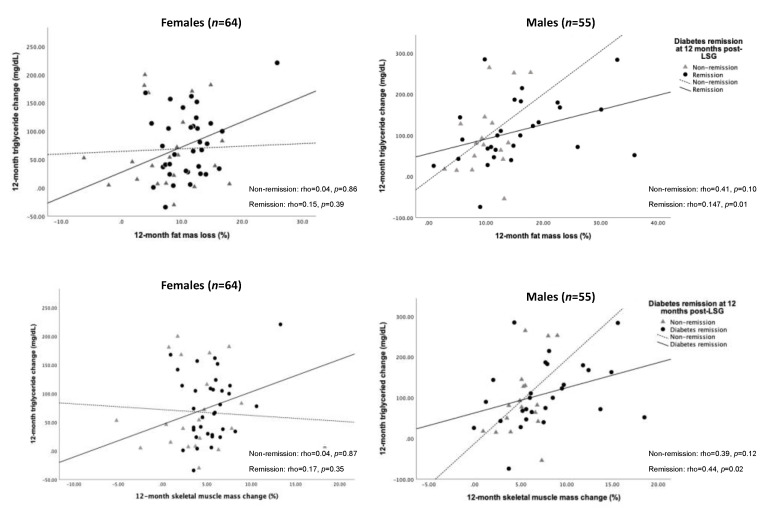
Spearman’s correlation of changes in FM loss, SM gain, and triglyceride over 12 months after LSG according to sex difference. Abbreviation: FM, fat mass; SM, skeletal muscle mass; LSG, laparoscopic sleeve gastrectomy.

We further tested the association between changes in TG level and FM loss and SM gain. As a result, the decreased TG level was significantly correlated with FM loss (rho = 0.47, *p* = 0.01) and SM gain (rho = 0.44, *p* = 0.02) for 12 months post-operation in males who achieved T2D remission (Figure 4).

## 4. Discussion

More than half of the patients with T2D and obesity achieved complete diabetes remission for 12 months after LSG in our study that was in line with the T2D remission rate after bariatric surgery from previous research [21]. Moreover, WL and FM loss were positively associated with diabetes remission in T2D patients with obesity one year after LGS. The higher proportions of FFM and SM gain were positively associated with the increased T2D remission in males, lacking evidence in females. 

LSG leads to sustainable WL, mainly occurring in the first postoperative year, and is stable in the following years [10]. As expected with WL, the observed FM loss in our study is higher than that shown in a previous study (11.3% vs. 10.8%) at 12 months after LSG [14]. Regarding weight gain and excess fat accumulation linking to T2D development [22], the greater the long-term WL was sustained, the higher percentage of diabetes remission was obtained. Our results confirmed that WL and FM loss were significantly higher in the diabetes remission group than in the non-remission group for 12 months post-LSG, which could reverse the diabetes process [22]. Furthermore, Ramos-Levi et al. suggested that when the 12-month percentage WL was added in the predictive model, it increased the correction of T2D remission in 95.9% of the cases at 12 months after bariatric surgery [23]. In agreement with previous findings [23], WL and FM loss should be considered the follow-up predictors of diabetes remission post-LSG. In terms of FM loss, removing excess fat from the liver mainly attributed to TG can enhance glycemic control [3,24]. In the present study, the FM loss was positively associated with the decreased TG level among the diabetes remission group 12 months after LSG (Figure 4). An increased plasma TG is one of the critical components of diabetic dyslipidemia [25], which is an important and common risk factor for coronary heart disease (CHD), which is the leading cause of morbidity and mortality worldwide [26]. The complex nature of diabetic dyslipidemia was linked to atherogenic lipid and lipoprotein abnormalities. By itself, atherogenic dyslipidemia characterized by high levels of TG-rich lipoproteins and their cholesteryl ester-rich remnant particles, low levels of HDL-cholesterol, and small dense LDL was associated with insulin resistance [25]. Notably, though a close positive correlation between fasting plasma TG and liver fat quantitated by using proton spectroscopy in humans was observed, the amount of fat in the liver is the cause, or the consequence of insulin resistance remained controversial. It is highly relevant to dyslipidemia with fat accumulation in the liver and insulin resistance may also predispose to the development of T2D [27]. 

A protein-enriched diet is highly recommended for long-term FM loss after bariatric surgery [12]. Noteworthy, Gomes et al. [28] revealed that whey protein supplementation promoted FM loss in women with long-term weight regain after bariatric surgery. Women who received whey protein (0.5 g/kg of the ideal body weight (IBW)/day) presented significantly more WL, accounting for FM loss and no change in FFM compared to the control group. In addition, the amount of dietary protein ≥ 1.1 g/kg of ideal body weight/day was recommended to prevent FFM loss for bariatric surgery patients [29]. Herein, the adequate amount of protein intake and protein supplementation is an efficient nutritional intervention that may promote more significant FM loss with FFM preservation. 

FFM and SM maintenance is crucial in weight reduction because of their role in metabolic regulation, skeletal integrity, and functional capacity. In line with previous findings [10], our FFM and SM expressed as percentages of the total body weight at 12 months post-LSG were higher in males than in females and higher in males with diabetes remission. In general, concerning metabolic rate, males naturally have more significant amounts of testosterone in their bodies, less body fat, and more muscle that leads to a higher metabolic rate than females [30,31]. The percent FFM loss of females and males was 22.9% and 26.1%, respectively that were considered as “safe” reduction for obese individuals ranging from 20–30% [32]. FFM and SM loss was expected with WL in the first post-bariatric surgery year [10] and the decreased SM was associated with obesity and T2D development [33]. One more important factor linking WL and body composition changes after Roux-en-Y gastric bypass (RYGB) is resting energy expenditure (REE). Lower postoperative REE is undesirable as it may contribute to less weight reduction or even long-term weight regain [34,35]; however, we could not assess this issue due to the nature of this retrospective study. Therefore, the association between FFM loss and REE is warranted in further studies. 

Meanwhile, the increase in low-density muscle or lipid-rich skeletal muscle, which includes fat components between and inside muscle fibers was significantly linked to insulin resistance in T2D patients with obesity [36]. Of note, a significant correlation between SM gain and TG reduction was observed in the present study. Much evidence has concluded that WL decreases the content of TG within SM, namely intramyocellular lipid (IMCL), which could improve insulin action and perhaps contribute to diabetic remission [37,38]. However, the conspicuous understanding of the mechanism in SM substrate metabolisms, such as defects of fatty acid metabolism centered at the mitochondria in obesity and T2D, has been unknown [8,39]. Herein, it is necessary to investigate the effect of changes in different muscle types (type I and II fibers) of FFM and SM on diabetes remission 12 months after LSG. Furthermore, muscle strength was impaired in patients with T2D and obesity [33,40]. Intriguingly, individuals who suffered from nutritional deficiencies such as vitamin D and B vitamins presented impaired muscle function [41]. Deficiencies of iron, 25OHD, and vitamin B12 were more frequently reported in patients after bariatric surgery while these patients adhered to prescribed supplements [42]. Hence, further study is required to investigate the role of nutritional deficiencies on the association of FFM and SM with diabetes remission. In addition, the improvement of SM strength and muscle insulin sensitivity can be achieved through resistance exercise [11], and males may do more high-intensity physical activities than females [43]. As is known, resistance training (RT) improves body composition by reducing body weight and FM while increasing FFM in individuals with obesity [44]. Interestingly, Lamarca et al. [44] found that the combined RT and adequate protein intake via whey protein supplementation for 12 weeks increased FFM and SM in patients 2–7 years after bariatric surgery without changing REE. Recent evidence showed that SM protects metabolically healthy boys but not girls with obesity from cardiometabolic risks [13]. It is conceivable that FFM and SM were well-maintained in the first postoperative year and decreased slightly from 1 to 5 years after bariatric surgery [10]. Thus, the nutritional intervention and RT can preserve FFM and SM at the end of the first post-surgical year, which is critical for a long-term diabetes remission post-LSG.

This study has several limitations. First, many necessary nutritional blood tests (such as vitamin D and B vitamins) were missing due to the nature of the retrospective cohort study. Though all patients were consulted to have specific diets with 80–90 g high-protein powders every day and exercise 45–60 min for 5–7 days per week, we did not assess physical diet status. Second, BIA was not a gold standard to estimate body composition; but it was accepted in research related to obesity [45]. Third, we did not assess the intracellular TG level in SM to explore its relationship with T2D remission post-LSG. Finally, we look forward to validating our findings in a larger study population with longer follow-up duration. Fourth, we did not calculate sample size before the study, so that negative female results cannot be generalized per se.

## 5. Conclusions

LSG is an efficient metabolic surgery for patients with T2D and obesity. LSG brings in massive WL, mainly focused on FM loss to improve insulin sensitivity. Preservation of FFM and SM in the first postoperative year is critical to diabetes remission in males but, at least in the present study, lacks evidence for females. Reducing the excess TG accumulation in SM by doing high-intensity physical activities and avoiding nutritional deficiencies can mitigate obesity and T2D complications.

## Figures and Tables

**Table 1:** Baseline demographic and disease characteristics.

Characteristic	Females (*n* = 64)	Males (*n* = 55)	*p*-Value
Age (yrs)	42.6 (9.5)	41.1 (9.4)	0.35
BMI (kg/m^2^)	41.4 (6.4)	43.3 (7.4)	0.17
Body weight (kg)	107.2 (18.2)	129.8 (25.4)	<0.01
Height (cm)	161.1 (0.1)	172.8 (0.1)	<0.01
FFM (kg)	55.4 (8.2)	74.6 (12.9)	<0.01
FFM (%)	52.0 (4.3)	57.9 (6.4)	<0.01
SM (kg)	30.8 (5.0)	42.2 (7.7)	<0.01
SM (%)	29.6 (2.4)	34.2 (4.1)	<0.01
FM (kg)	51.8 (12.0)	55.6 (16.8)	0.22
FM (%)	47.9 (4.3)	29.3 (8.4)	<0.01
FBG (mg/dL)	172.0 (70.1)	212.5 (84.6)	0.01
HbA1c (%)	7.7 (1.7)	8.2 (1.6)	0.11
TC (mg/dL)	193.1 (44.5)	184.7 (37.2)	0.26
LDL-C (mg/dL)	126.7 (41.8)	123.2 (35.9)	0.63
TG (mg/dL)	193.1 (44.4)	184.7 (37.2)	0.13

Data presented as a mean (SD). Abbreviation: BMI, body mass index; FBG, fasting blood glucose; FFM, fat-free mass; FM, fat mass; HbA1c, glycated hemoglobin; LDL-C, low-density lipoprotein cholesterol; SM, skeletal muscle mass; TC, total cholesterol; TG, triglyceride.

**Table 2:** Body composition changes at 12 months after LSG according to sex.

Variables	Females (*n* = 64)	Males (*n* = 55)	*p*-Value ^#^
BMI (kg/m^2^)	−10.4 (3.8) *	−12.1 (4.8)	0.04
Body weight (kg)	−27.1 (9.9) *	−36.4 (14.4) *	<0.01
Weight loss (%)	−24.9 (7.1)	−27.7 (8.7)	0.07
FM (kg)	−20.9 (8.0) *	−27.0 (13.1) *	0.01
FM (%)	−9.9 (5.0) *	−12.8 (6.2) *	0.03
FFM (kg)	−6.2 (4.1) *	−9.5 (6.4) *	0.01
FFM (%)	+9.9 (5.0) *	+12.8 (8.0) *	0.03
SM (kg)	−3.9 (2.7) *	−6.2 (3.9) *	0.01
SM (%)	+4.9 (6.2) *	+6.5 (4.3) *	0.04

Data presented as mean difference (SD), indicating the changes of body composition after one year post-surgery. * *p* < 0.05 presents significant mean differences (MD) of covariates at baseline and at 12 months post-LSG using a paired student’s *t*-test. ^#^ A student’s *t*-test was used to compare the mean of body composition changes between females and males. Abbreviation: FFM, fat-free mass; FM, fat mass; LSG, laparoscopic sleeve gastrectomy; SD, standard deviation; SM, skeletal muscle mass.

**Table 3:** Association between body composition changes and type 2 diabetes remission at 12 months post-LSG.

12-Month Post-LSG	Total Participant ^a^		Females (*n* = 64) ^b^		Males (*n* = 55) ^b^	
	OR (95% CI)	*p*-Value	OR (95% CI)	*p*-Value	OR (95% CI)	*p*-Value
WL (%)	1.13 (1.05–1.22)	<0.01 *	1.11 (1.01–1.23)	0.04 *	1.14 (1.03–1.25)	<0.01 *
FM loss (%)	1.14 (1.04–1.26)	<0.01 *	1.15 (1.02–1.31)	0.03 *	1.16 (1.03–1.31)	0.01 *
FFM gain (%)	1.14 (1.04–1.26)	<0.01 *	1.09 (0.95–1.24)	0.21	1.16 (1.02–1.32)	0.02 *
SM gain (%)	1.16 (1.01–1.34)	0.04 *	1.02 (0.84–1.25)	0.83	1.26 (1.02–1.55)	0.03 *

^a^ Adjusted for sex, age (years), and baseline FBG (mg/dL). ^b^ Adjusted for age (years) and baseline FBG (mg/dL). * *p* < 0.05. Abbreviation: CI, confidence interval; FBG, fasting blood glucose; FFM, fat-free mass; FM, fat mass; LSG, laparoscopic sleeve gastrectomy; OR, odd ratio; SM, skeletal muscle mass; T2D: type 2 diabetes; WL, weight loss.

## Data Availability

The data used in this manuscript are fully disclosed in the Tables of the manuscript and are available on request from the corresponding author.
